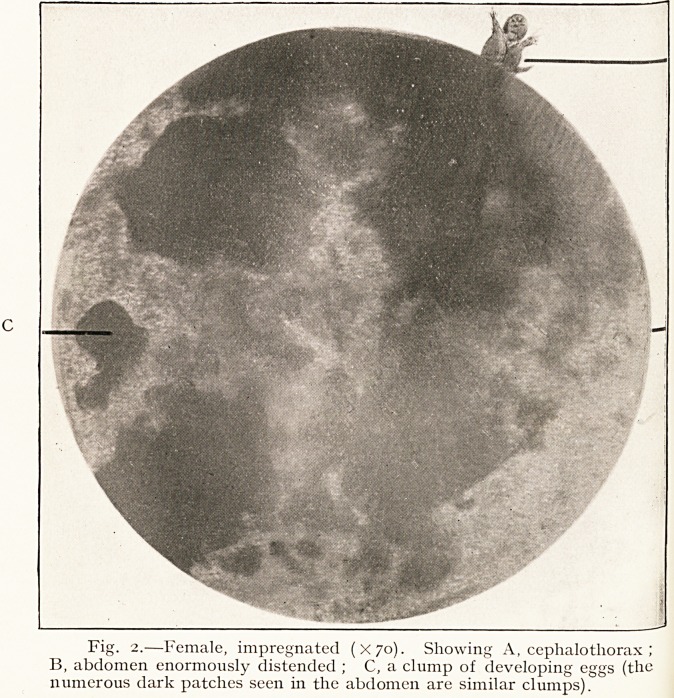# Cotton-Seed Dermatitis and Its Cause, Pediculoides Ventricosus

**Published:** 1915-06

**Authors:** J. A. Nixon

**Affiliations:** Captain, R.A.M.C.(T.), Second Southern General Hospital; Physician in Charge of Skin Department, Bristol Royal Infirmary; Clinical Lecturer in Dermatology, University of Bristol


Fig. 1.—Male (×44). Ventral surface, showing sclerites at base of limbs. Note that first and last pair of appendages are clawed.

Fig. 2.—Female, impregnated (×70). Showing A, cephalothorax; B, abdomen enormously distended; C, a clump of developing eggs (the numerous dark patches seen in the abdomen are similar clumps).

LIBRARY OF CONGRESS
AUG 31 1915
SMITHSONIAN DEPOSIT.

The Bristol

Medico-Chirurgical Journal.

"Scire est nescire, nisi id me
Scire alius sciret."

JUNE, 1915.

### COTTON-SEED DERMATITIS AND ITS CAUSE, PEDICULOIDES VENTRICOSUS.

BY

J. A. Nixon, M.B. Cantab., F.R.C.P.,

*Captain, R.A.M.C.(T.), Second Southern General Hospital;*
*Physician in Charge of Skin Department, Bristol Royal Infirmary;*
*Clinical Lecturer in Dermatology, University of Bristol.*

---

Some years ago Dr. Kenneth Wills[1] published an account of an outbreak of "barley itch" amongst some of the dock labourers in Bristol engaged in discharging cargoes of barley. A full description of the disease is given (with a good bibliography) in Stelwagon's *Diseases of the Skin,*[2] and Shipley[3] refers briefly to it in his *Minor Horrors of War.*

The eruption in "barley itch" is believed to be due to the bite of a "mite" called *Pediculoides ventricosus.*

[1] *British J. Derm.*, Aug., 1909.

[2] Stelwagon, *Treatise on Diseases of the Skin*, Seventh Edition, 1914, p. 1183.

[3] Shipley, *Minor Horrors of War*, 1915.

An outbreak of a somewhat similar nature has recently been the subject of investigation, occurring at the Avonmouth Docks amongst dockers who handled cargoes of cotton seed. The Medical Officer of Health for Bristol, Dr. D. S. Davies, first drew my attention to this outbreak at the end of 1914, and the Health Office endeavoured, without success, to find a patient who displayed the rash in an early stage and unattended by scratching and secondary infection.

In January, 1915, however, the Secretary of the Dockers' Union was able to bring under my observation a man whose eruption had only just appeared.

T. T., a healthy dock labourer, aged 42, had enjoyed good health all his life, and had only drawn six weeks' sick pay from his club in fourteen years. He was not liable to food rashes, and presented no sign of scabies or body-lice. His history of the present condition was that three days before seeing the exhibitor he had been at work unloading a cargo of cotton seed (in bulk) from Alexandria. Within a short time of starting work on the cotton seed the patient began to feel some irritation about the neck and arms. This irritation increased, and became most severe during the following night when he got warm in bed. At first no rash could be seen, but towards evening a series of red spots, about the size of mosquito bites, appeared at the site of the irritation. Some of the spots developed " bladders " upon them, which burst and discharged a watery fluid. He had previously had similar attacks, the first occurring some four or five years ago, but the present attack was the worst he had suffered from. The patient stated that he had developed a somewhat similar eruption from working in " itchy " barley.

The rash died out in a week if not renewed by continued work in the " cotton seed," or unless it was " scratched and poisoned," when a " sort of eczema set in." The rash did not appear on the covered parts of the body. The spots were not transient or recrudescent. There must be actual contact with the seed before the itching started ; mere entrance into the place where the seed was stored did not cause any itching. It was only certain cargoes of cotton seed which were " itchy " ; the men thought that the " itchy " cargoes were those which came from Alexandria ; there had been no complaints with those from Smyrna. But cotton seeds if in bags did not seem harmful ; it was only when handling cotton-seed cargoes " in bulk" that the "itch" occurred. Of fifty men working on this cargo about two-thirds had been attacked.

The eruption consisted of sparsely-distributed, isolated urticarial papules situated chiefly on the neck and forearms with a few papules on the legs. Each papule was pinkish-red in colour, hard, raised and about the size of a pea. In its general appearance the rash resembled a moderately severe attack of lichen urticatus in a child. There were no burrows of the *Acarus scabiei* to be seen.

Fortunately I was able to get fresh supplies of the cotton seed in which numerous live "mites" were readily seen under the low power of the microscope. These resembled fairly closely the *Pediculoides ventricosus* as figured in Stelwagon, although no examples of the gravid female (from which this mite derives its distinguishing name) could be found. The cargo had only recently been discharged from the hold, and probably offered a warm habitation to the insects, as three days' exposure of a small sample to the cold left none of them alive.

It appears that the "mites" do not tend to become parasitic on man. None of the workers who were attacked complained of being bitten afresh in their own homes, nor did the disease spread to any members of their families.

Dr. MacLeod,[1] in discussion at the Dermatological Section of the Royal Society of Medicine, expressed the opinion that these "mites" of cotton-seed dermatitis were parasites of a caterpillar of the cotton moth (*Gelechia gossypiella*), which he and Colonel Alcock had found present in a cargo of cotton seed that had caused a similar dermatitis at the London Docks about eighteen months previously.

In the first specimens of cotton seed from the Avonmouth and Bristol Docks no traces of this moth or its caterpillar were discovered by Dr. Henderson. In the later samples, however, the caterpillar was frequently

[1] *Proc. Roy. Soc. Med.*, 1915, viii. 112.

discovered coiled up in a hollow seed which it had eaten out. In these samples many gravid females were found, proving conclusively that the "mite" is the *Pediculoides ventricosus.* The illustration given in Stelwagon (from Laboulbène and Mégnin) does not quite bring home the extraordinary disproportion in size between the non-gravid and the gravid female.

Dr. W. D. Henderson, Lecturer in Charge of Department of Zoology, University of Bristol, has kindly examined and reported on the cotton seed and the "mite" found in it.

The following account is based on material obtained from cotton seeds taken from the S.S. *Andromachis* :—

The cotton seeds were examined immediately on arrival in the laboratory, and were found to be richly infested with the "mite." They were moving about in a rather lethargic way, and a number of preparations were made.

In order to see if heat had any effect on the specimens the seeds were collected in a sample bag, and placed in an incubator which had a constant temperature of 33° centigrade. Exactly twenty-four hours afterwards the seeds were again examined, and instead of the slow movements of the "mites" previously noticed, they were now found to be vigorously and rapidly moving on the slide, and it was from this material that the best specimens were obtained.

A number of living and active specimens were selected under the binocular microscope and placed in a large drop of 50 per cent. glycerine at 10.0 a.m. on June 18th, and were examined twice daily for twelve days. At 5.0 p.m. on the twelfth day they were still moving their legs fairly actively at times, and at other times lying quite still. On the thirteenth day I was unable to examine them, but at twelve noon on the fourteenth day they were quite motionless. The glycerine, as far as I could judge by testing with litmus paper, was neutral.

This suggests that we are dealing with a highly-resistant form, and one that is likely to resist all attempts at treating the cargo in bulk with an insecticide which would be effective, and at the same time possible on the score of expense.

The animal is elongated and flattened dorso-ventrally, with an average length of 0.16 mm. and an average breadth of 0.062 mm. It is sharply pointed at the anterior end and more rounded at the posterior in the female, more truncated in the case of the male.

The impregnated female often reaches a length of 1.3 mm. This increase is entirely due to the large swollen globular abdomen, which varies in diameter from 0.8 mm. to 1.5 mm. Several of the specimens reached a length of 1.6 mm., of which the swollen abdomen was fully 1.5 mm. in length.

There are four pairs of walking legs on which there are bristles, which are apparently more numerous on the female than on the male.

The body of the animal is soft, but there are several sclerites present, especially on the ventral surface at the points of insertion of the appendages.

On the dorsal surface three shallow transverse grooves are visible, thus dividing the body into four distinct regions. On the ventral surface the body is marked out into a number of regions, one fairly large quadrilateral region at the base of each of the four anterior walking legs. These are separated off from one another by a median groove which does not quite reach the posterior margin of the hinder area of the two, but at its anterior end it bifurcates and curves gradually round till each arm reaches the margin, thereby separating off a small area which lies at the base of the pedipalps.

At the base of each of the four posterior legs there is also a distinct area marked off, but these differ in shape from the areas associated with the anterior walking legs. At the base of each of the third pair there is a large, more or less, oval-shaped area, which does not meet its fellow in the middle line. Each of the fourth pair has a much smaller triangular-shaped area, which also does not join its fellow. Behind this there is a partially differentiated area on each side which can be traced about half-way towards the middle line.

The areas at the base of the third and fourth pairs of walking legs show a tendency to be divided up into a median and a smaller lateral area.

The cephalothorax is marked off from the abdomen, but their line of demarcation is by no means very distinct.

The two anterior pairs of walking legs rise fairly closely together, just behind the basal ends of the mouth parts. Then there is a considerable gap, and the two posterior pairs rise close together a short distance from the posterior end of the animal.

The walking legs are six-jointed. The first pair tapers less than the others, and differs slightly in the distal joint, which is haired and clawed. The remaining pairs are longer and more slender, and the tarsal joint is much narrower than the other joints, and has a peculiar cone-shaped swelling at its end.

About half-way up the tarsal joint there is a pair of peculiar lateral outgrowths which gives the tarsus a †-shaped appearance.

In the males the last pair of walking legs is also clawed.

The cheliceræ are apparently reduced to stylet-like structures which are capable of protrusion. The pedipalps are also greatly modified and partially fused with the maxillary plate, and terminate in a hard, clawed lip.

There are two well-marked tracheæ which are coiled and arranged more or less symmetrically near the margins of the body. There is near the base of the first pair of walking legs a peculiar structure which, running obliquely forward on each side, reaches the exterior near the base of the pedipalps. This in our previous account was considered as either a modified anterior portion of the tracheæ, as by focussing at different levels it seemed to be continuous with the tracheæ, or as the pseudo-stigmatic organs. While still doubtful as to the real nature of these structures, it seems more probable that they are the pseudo-stigmatic organs or a modification thereof.

The internal structure, as far as can be made out, does not differ to any extent from the published account given in most text-books.

The hairs on the body and appendages :—

(*a*) On the body there are usually five pairs of hairs. Four pairs are borne on the thoracic portion and one pair rises close to the posterior end of the abdomen. Occasionally there are fewer pairs visible, but this may possibly be due to the specimens being damaged in the handling of the cargoes from which the material was obtained. One point of considerable interest is that in none of the pregnant females can I find any trace of the last pair of long hairs which rise near the posterior end of the abdomen in the males and non-pregnant females.

(*b*) In the female on each joint of the walking legs there is a pair of stiff hairs. The first pair of walking legs has a number of hairs on its distal joint.

In the male the hairs on the joints of the walking legs are shorter and more slender, but in addition to these there is on each walking leg a pair of longer and stouter hairs. These hairs, which are so conspicuous on the first pair of walking legs, are present only on one side of the limb, and arise one from each of two adjacent joints.

The distal joints of the first pair and of the last pair of walking legs are similar to those joints in the first pair of appendages of the female.

There is one fact in connection with *Pediculoides* that is too often overlooked, namely that both in its young and adult stages it lives on the larvæ of different insects. This is fully brought out by the present material, as the most abundant supply of specimens was obtained from seeds the interior of which had been destroyed by a caterpillar-like larva. These larvæ were unfortunately dead, and in most cases badly damaged, so that they could not be run down to any group, but it is quite possible that they may be the larvæ of the cotton moth, as Dr. MacLeod suggests.

The classification of the *Acarina* is in a very unsatisfactory state, although well-known arachnologists have tried to reduce it to some order. With regard to the affinities of the various groups widely divergent views are held. a state which is not at all conducive to any definite result being reached in the classification. More unfortunate still is the fact that there is little agreement among the workers as to the relative value of the characters upon which the classification is based. The result is that one authority gives thirty-four families, while another equally important authority gives only ten.

The specimen in question, *Pediculoides ventricosus*, belongs to the *Tarsonemidæ*, and is placed in a special sub-order of its own on the one hand, on the other it is classed along with the *Trombidiidæ*.

It is probable that for some considerable time yet it will be most satisfactory from the practical point of view to draw up a purely artificial classification which will have less than thirty-four and more than ten families. If this is done, the increasing importance of these *Acarina*, both from the medical and the legal aspect, will no doubt set some workers going to decide which are harmful and which are not. This is the type of work that will eventually lead to a proper classification.

Dr. A. E. Shipley having expressed his interest in this new "minor horror," kindly submitted a specimen to Mr. C. Warburton, of Cambridge, who writes of it thus :—

"I think Dr. Nixon may have got the culprit. It is so excessively small that unless alive it would be very hard to find. His mite is one of the Tarsonemidæ. Banks[1] quotes Karpelles as saying of a mite of the same group infesting barley in Russia, 'The men had been handling barley, and the mites spread from this to the hands, when they caused an irritating inflammation of the skin so intense as to force the men to leave their work.'"

In May, 1915, another outbreak of cotton-seed dermatitis was reported to me by Dr. D. S. Davies, who had been

[1] *Treatise on the Acarina*, p. 77.

consulted by Dr. W. M. Hope, Medical Officer of the Port of Gloucester.

In June, 1915, I had the opportunity of visiting two steamers in the Bristol Docks where cargoes of cotton seed were discharging. The cotton seed was in bulk in the hold, and nearly all the men working on it were severely bitten on the arms and neck. The eruption was quite uniform, and of papulo-urticarial nature, resembling that described earlier in this paper in the case of the man T. T. I was on board the steamers for a considerable time with cotton seed all round and strewn about the deck, but as I refrained from handling the cotton seed I escaped without being attacked with any irritation, and apparently without being bitten.

The men engaged on these cargoes told me that they had noticed a peculiarity in the "itchy" cotton seed which was new to them, and in their minds was the cause of the "itchiness," namely, that a very large number of the seeds were hollow, and contained a worm or maggot. Up to the present I cannot trace any connection between the maggot and the mite, unless Dr. MacLeod's suggestion is the correct one that the *Pediculoides ventricosus* is a parasite of the caterpillar of the cotton moth, and these "maggots" which the men referred to are in reality this caterpillar.

The occurrence of these minor disablements is of increasing importance since the passing of the National Insurance and the Workmen's Compensation Acts.

The working man attacked by cotton-seed dermatitis claims that he is incapacitated by an injury or disease caused by or arising out of his employment.

The dermatitis itself is no great disablement, but a secondary infection or resulting eczema may prove long-lasting and intractable.

In the interest both of the labourer and the employer it is advisable to render work of this sort innocuous. It is not yet clear how this should be achieved, but the suggestion that dermatitis does not occur when the cotton seed is packed in bags is worthy of attention.

The cotton-seed mite seems to require a considerable degree of warmth, and quickly succumbs to exposure to the cold of an English January.

The illustrations in this article are from actual photomicrographs taken by Dr. Henderson, who, in spite of great difficulties, has obtained exceptionally good representations of *Pediculoides ventricosus*.

---

## Figures and Tables

**Fig. 1. f1:**
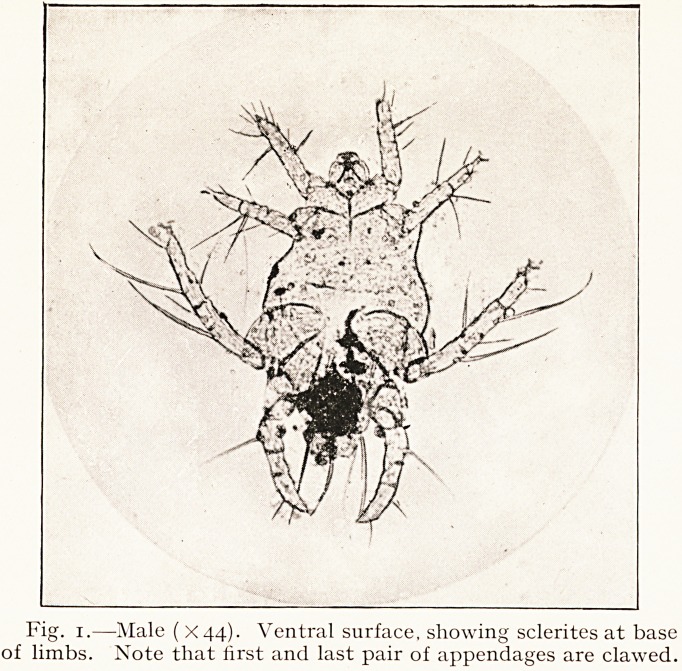


**Fig. 2. f2:**